# Three Consecutive Cases of Ocular Polyhexamethylene Biguanide (PHMB) Toxicity Due to Compounding Error

**DOI:** 10.7759/cureus.38540

**Published:** 2023-05-04

**Authors:** Nikunj V Patel, Umang Mathur, Sanil Sawant, Manisha Acharya, Arpan Gandhi

**Affiliations:** 1 Department of Cornea, Dr. Shroff's Charity Eye Hospital, New Delhi, IND; 2 Department of Laboratory Services, Dr. Shroff's Charity Eye Hospital, New Delhi, IND

**Keywords:** polyhexamethylene biguanide, toxicity, acanthamoeba keratitis, cataract, persistent epithelial defect

## Abstract

Acanthamoeba keratitis is treated with long-term biguanide therapy, and the treatment itself can lead to ocular side effects. Knowledge of possible toxic complications can help in the better titration of the treatment regimen. Here, we describe the toxic side effects of polyhexamethylene biguanide (PHMB), which occurred in three consecutive patients treated with in-house compounded PHMB. There was an error in compounding the solution, with the resultant concentration of PHMB being around 0.2%. Patients developed ocular toxicity like conjunctival inflammation, persistent epithelial defect, and large pigment clumps on endothelium within six weeks of initiation of therapy. All of them developed rapidly progressive cataract and mydriatic pupil within three months. PHMB has the potential to cause irreversible damage to ocular structures, and the toxicity is time and concentration-dependent.

## Introduction

Acanthamoeba keratitis is difficult to treat because of the innate properties of the organism to evade host defenses. Anti-Acanthamoeba agents are prescribed at a higher frequency, usually hourly, which can promote encystation as a defensive mechanism [1]. For these reasons, treatment must continue for many months [2]. Cationic biguanides, polyhexamethylene biguanide (PHMB), and chlorhexidine are considered first-line therapy. Both are equally efficacious and given either in combination with diamidines or as monotherapy [3]. Biguanides are unavailable commercially as eye drops and are prepared from 20% pharmaceutical stock solutions. Both are generally safe and well tolerated in the range of 0.02-0.06%; however, data on the ocular toxicity of PHMB is scarce compared to chlorhexidine. Corneal side effects like punctate keratopathy with usual concentration; edema, ulceration, and delayed healing with 4% concentration [4]; irreversible damage, including limbal stem cell deficiency (LSCD), endothelial loss, cataract, and glaucoma with 20% concentration have been reported previously with chlorhexidine [5,6]. We report three consecutive cases of PHMB toxicity occurring while treating Acanthamoeba keratitis. Written informed consent was obtained from all the cases for the purpose of publication.

## Case presentation

**Figure 1:**
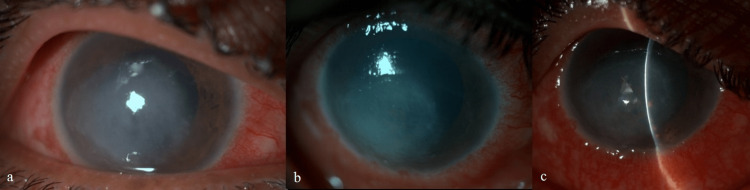
Images of Case 1 (a) at presentation, (b) after two weeks of polyhexamethylene biguanide (PHMB) treatment, (c) after six weeks of treatment

A 35-year-old male farmer presented with complaints of redness, watering, and diminution of vision in the right eye for three weeks. He was initially diagnosed as a case of viral keratitis and later as fungal keratitis elsewhere. At the presentation, he was on topical therapy of antifungal, antibiotic, and cycloplegic eye drops. There was no history of trauma or exposure to contaminated water. On examination, the best corrected visual acuity (BCVA) was hand movement perception in the right eye and 20/20 in the left eye. Slit lamp bio-microscopy of the right eye showed conjunctival congestion, 6x7 mm corneal epithelial defect with underlying dense mid-stromal infiltrate measuring 6x6 mm, and underlying moderate Descemet membrane folds (Figure 1a).

The anterior chamber was quiet with a clear lens; fundus details were indiscernible in the right eye. Diagnostic corneal scraping revealed Acanthamoeba cysts on a 10% potassium hydroxide (KOH) mount and calcoflour-white (CFW) stain. The patient was started on 0.02% polyhexamethylene biguanide (PHMB) eye drops once hourly, 2% homatropine hydrobromide eye drops three times a day, and 0.5% carboxymethyl cellulose eye drops four times a day in the right eye. PHMB drops were compounded in the hospital pharmacy from 20% PHMB pharmaceutical solution (Sigma Laboratories Pvt. Ltd., Mumbai, India).

There was a significant improvement in the patient's symptoms and the size of the infiltrate by day 3. On day 12, culture on non-nutrient agar (NNA) showed Acanthamoeba cysts. After six weeks of topical anti-Acanthamoeba treatment, the right eye cornea showed a healing infiltrate, a 5x4 mm large persistent epithelial defect (PED) with superficial vascularization, and large pigment clumps on the endothelium (Figure 1c). The pupil was dilated and fixed. At this point, PHMB toxicity was suspected, and the patient was advised to stop PHMB eye drops, and topical 0.1% fluorometholone eye drops were added four times per day. At ten weeks follow-up, he was advised the right eye tarsorrhaphy for non-healing PED. Subsequently, he also underwent amniotic membrane grafting (AMG) for non-healing defect at three months follow-up. On his last follow-up, he still had a small PED and a total white cataract in his right eye.

**Figure 2:**
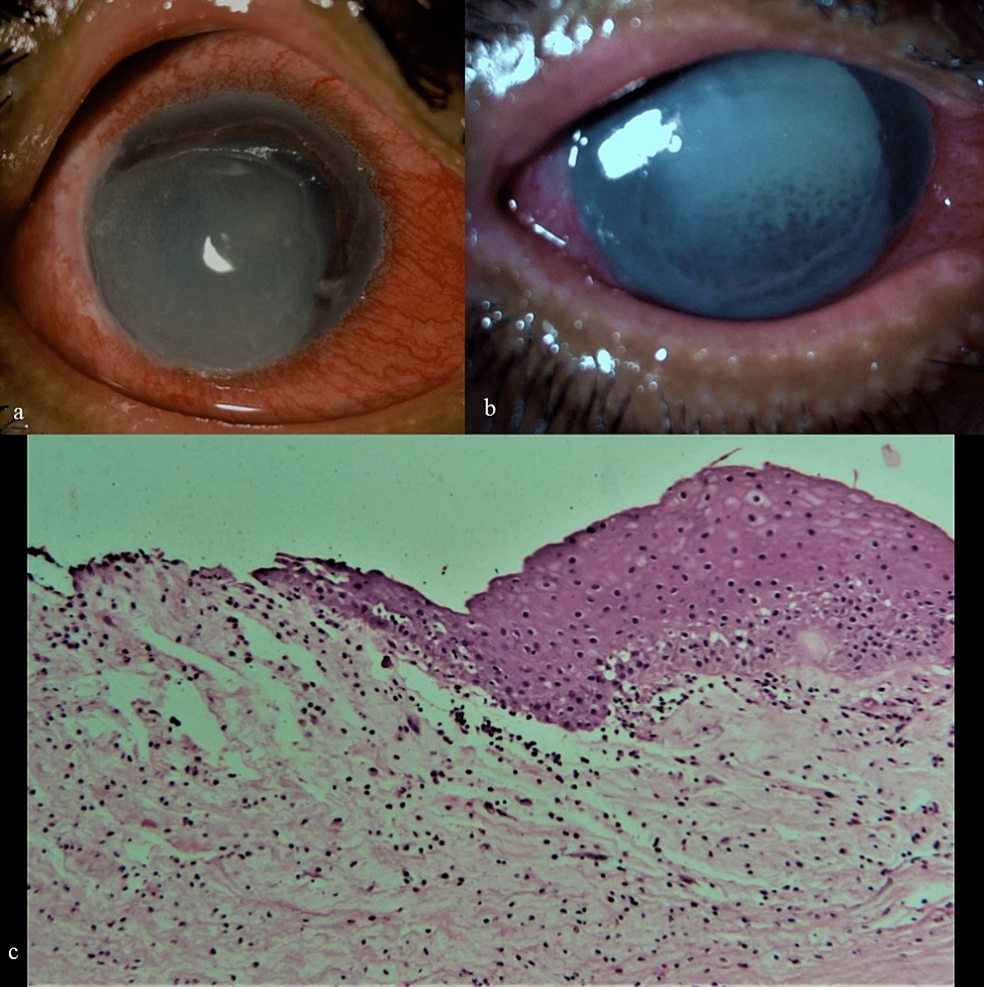
Images of Case 2 (a) at presentation, (b) pigments clumps on endothelium, mydriatic pupil, and total cataract six weeks after polyhexamethylene biguanide (PHMB) treatment, (c) histopathology of penetrating keratoplasty done five months later showing mild vascularization, mild chronic nonspecific inflammatory cells, loss of stromal keratocyte nuclei, stromal edema

**Figure 3:**
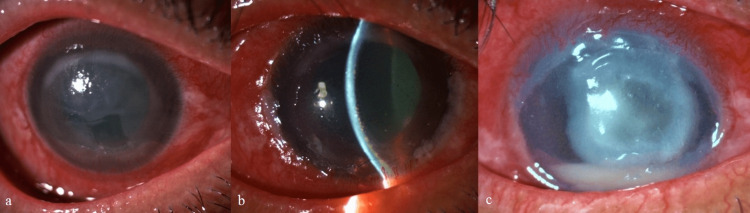
Images of Case 3 (a) at presentation, (b) pigment clumps on endothelium and mydriatic pupil five weeks later, (c) secondary fungal keratitis

Details of the other two cases are described in Tables 1-2 and Figures 2-3).

**Table 1:** Patient characteristics

	Case 1	Case 2	Case 3
Age (years)	35	48	49
Gender	Male	Male	Male
Eye	Right	Right	Right
Acanthamoeba keratitis	Smear and culture positive	Previous smear positive	Smear positive
Duration of polyhexamethylene biguanide (PHMB)	Six weeks	Six weeks	Five weeks
Frequency of PHMB	Once an hour the first week; twice an hour for five weeks	Once an hour the first week; twice an hour for five weeks	Once an hour the first week; twice an hour for four weeks
Persistent epithelial defect	Present	Present	Present
Absence of pain	After two weeks	After two weeks	After three weeks
Pigment clumps on endothelium	After six weeks	After six weeks	After five weeks
Cataract	Present	Present	Present
Mydriatic pupil	Present	Present	Present
Additional surgery	Tarsorrhaphy, amniotic membrane grafting	Tarsorrhaphy, penetrating keratoplasty with cataract extraction, Ahmed glaucoma valve surgery, retinal detachment surgery	Tarsorrhaphy, penetrating keratoplasty with cataract extraction

All three cases were treated with compounded topical PHMB eye drops for non-contact lens-related Acanthamoeba keratitis. Patients belonged to different geographical areas. All of them had a good clinical response in the initial period of intensive therapy, which correlated with the symptomatic relief of pain and a decrease in the size of corneal infiltration. However, five to six weeks later, they all started developing signs of toxicity in the form of an inflamed ocular surface, large PED, corneal edema, and characteristic pigment dusting of the endothelium. In addition, they had a significant absence of corneal melting, and all developed total cataract and mydriatic pupil within two to three months.

## Discussion

Polyhexamethylene biguanide hydrochloride (polyhexanide, PHMB) is a chemical biocide and a member of the polymeric guanidine family. It is used as a general disinfecting agent in the food industry, for contact lens solutions, and for the disinfection of swimming pools. It has an extremely low aggregate risk of adverse health effects on the public or environment, except for occupational users. It has been in use as a surface disinfectant and as a safe and nontoxic anti-infective agent in wound management for near about 30 years. It has been in use for treating Acanthamoeba keratitis since 1992 after Larkin et al. conducted in vitro and clinical studies and determined 0.02% concentration to be effective against Acanthamoeba [7]. Anterior segment toxicity of chlorhexidine has been reported earlier. In our case series, we describe three cases where patients developed side effects to PHMB similar in spectrum to chlorhexidine toxicity but occurred in a shorter period of duration. 

Cataract development and iris atrophy have been described previously in patients treated for Acanthamoeba keratitis [8,9]. Both case series used combination therapy with a standard dose of 0.02%, and side effects developed four to six months later.

Cysticidal and cytotoxic effects of anti-Acanthamoeba agents are concentration and time-dependent [10]. In vitro cell culture study conducted by Shi et al. showed that biguanides and diamidines both cause concentration and time-dependent cytotoxicity. PHMB decreases cell viability more than chlorhexidine [11]. Lee et al. also showed that PHMB is more toxic to keratocytes than chlorhexidine [10]. Mafra et al., however, reported that chlorhexidine monotherapy was more cytotoxic than PHMB and that combination biguanide therapy in the concentration of 0.04% may be less toxic than monotherapy [12].

Phase 1 clinical trial of Orphan Drug for Acanthamoeba Keratitis (ODAK) project showed that 0.02-0.08% PHMB is effective against Acanthamoeba polyphaga and has good ocular tolerability, with 0.04% concentration as the most effective dose. A concentrated 0.8% PHMB solution was found to have irreversible damage to the ocular surface. The results were replicated in ocular safety and tolerability study in healthy volunteers [13]. Rabbit studies have shown that a single application of 20% aqueous solution of PHMB produced iritis, conjunctivitis, and corneal opacity, all of which recovered by the 25th day, whereas neat PHMB caused irreversible damage at 21-day observation in the form of conjunctivitis, corneal opacity, and vascularization [14]. Lim et al. conducted a safety study in a rabbit model wherein they found that direct intrastromal injection of 0.02% PHMB resulted in corneal epithelial erosion, corneal edema, and severe neovascularization. However, 0.01% PHMB did not induce apparent corneal toxicity [15].

The toxicity that occurred within a short duration of use of topical PHMB in our case series could be because of a compounding error in dispensing PHMB; 0.1 ml of the 20% PHMB stock solution should be diluted in 100 ml of normal saline to prepare 0.02% eye drops. Written instructions were unavailable at the pharmacy, and we could not retrieve the stock solution from the pharmacy as it was discarded. On review, it was found that 1 ml of PHMB solution was taken, making it a 0.2 % solution. It was a limitation of our study that we could not test the stock solution for the presence of any other chemical which could have been added accidentally. However, cases treated before these three cases and those treated after with chlorhexidine monotherapy did not show similar features in the short term.

PHMB may act as a chemical corrosive agent, toxicity being concentration and time-dependent. PHMB may directly affect corneal and conjunctival cells, stromal collagen, keratocytes, corneal nerves, endothelium, and even iris and lens structures. Therapy is usually started when there is an epithelial defect. As the initial frequency is high, there is increased penetration of these cationic agents. There is evidence that these agents bind to proteins on cellular surfaces and are therefore released in a sustained manner. This causes progressive damage to the stromal collagen and keratocytes. Though not studied with regards to its effect on corneal nerves, we believe these agents do cause structural damage to unmyelinated corneal nerves, as evidenced by relief from pain despite large epithelial defect and ocular surface inflammation. Loss of keratocytes, damage to corneal nerves, and possible damage to limbal stem cells may contribute to progressive stromal ulceration, as seen in our cases. As the intrastromal concentration is high, diffusion in the anterior segment is possible, which can lead to chemical injury to the iris and lens.

It can be argued whether this presentation is due to drug toxicity or the immunological response to leftover antigens of the cysts. Studies have shown that Acanthamoeba trophozoites can cross Descemet's membrane but are countered by the neutrophils present in aqueous, thereby limiting intraocular spread [16]. In the literature, only four cases have been reported about intraocular spread, three post-penetrating keratoplasty, and one post-cataract surgery [17-20]. Intraocular dissemination appears to be a rare event and occurs most likely after surgical intervention. Also, serial follow-up on in-vivo confocal microscopy (IVCM) could have helped better understand the disease process but was not done because of nonavailability.

## Conclusions

As anti-Acanthamoeba agents are chemical disinfectants, an ocular response resembling chemical injury is highly likely in the event of chronic use of such medications. Until better therapeutics are available, judicious use of such agents is warranted. Acanthamoeba keratitis is difficult to manage largely because of its masquerading nature and limited treatment options. Treatment should include tolerable topical drug concentrations and appropriate spacing when using combination therapy. Clinicians should look out for signs of developing drug toxicity and discontinue the treatment, as it is likely to be irreversible if continued.

## References

[1] Turner NA, Russell AD, Furr JR, Lloyd D (2000). Emergence of resistance to biocides during differentiation of Acanthamoeba castellanii. J Antimicrob Chemother.

[2] Papa V, Rama P, Radford C, Minassian DC, Dart JK (2020). Acanthamoeba keratitis therapy: time to cure and visual outcome analysis for different antiamoebic therapies in 227 cases. Br J Ophthalmol.

[3] Lim N, Goh D, Bunce C, Xing W, Fraenkel G, Poole TR, Ficker L (2008). Comparison of polyhexamethylene biguanide and chlorhexidine as monotherapy agents in the treatment of Acanthamoeba keratitis. Am J Ophthalmol.

[4] Phinney RB, Mondino BJ, Hofbauer JD (1988). Corneal edema related to accidental hibiclens exposure. Am J Ophthalmol.

[5] Shigeyasu C, Shimazaki J (2012). Ocular surface reconstruction after exposure to high concentrations of antiseptic solutions. Cornea.

[6] Steinsapir KD, Woodward JA (2017). Chlorhexidine keratitis: safety of chlorhexidine as a facial antiseptic. Dermatol Surg.

[7] Larkin DF, Kilvington S, Dart JK (1992). Treatment of Acanthamoeba keratitis with polyhexamethylene biguanide. Ophthalmology.

[8] Ehlers N, Hjortdal J (2004). Are cataract and iris atrophy toxic complications of medical treatment of acanthamoeba keratitis?. Acta Ophthalmol Scand.

[9] Herz NL, Matoba AY, Wilhelmus KR (2008). Rapidly progressive cataract and iris atrophy during treatment of Acanthamoeba keratitis. Ophthalmology.

[10] Lee JE, Oum BS, Choi HY, Yu HS, Lee JS (2007). Cysticidal effect on acanthamoeba and toxicity on human keratocytes by polyhexamethylene biguanide and chlorhexidine. Cornea.

[11] Shi L, Stachon T, Seitz B, Wagenpfeil S, Langenbucher A, Szentmáry N (2018). The effect of antiamoebic agents on viability, proliferation and migration of human epithelial cells, keratocytes and endothelial cells, in vitro. Curr Eye Res.

[12] Mafra CS, Carrijo-Carvalho LC, Chudzinski-Tavassi AM, Taguchi FM, Foronda AS, Carvalho FR, de Freitas D (2013). Antimicrobial action of biguanides on the viability of Acanthamoeba cysts and assessment of cell toxicity. Invest Ophthalmol Vis Sci.

[13] (2020). Orphan drug for Acanthamoeba keratitis. https://cordis.europa.eu/project/id/305661/reporting..

[14] Bernauer U (2015). Opinion of the scientific committee on consumer safety (SCCS) - 2nd revision of the safety of the use of poly(hexamethylene) biguanide hydrochloride or polyaminopropyl biguanide (PHMB) in cosmetic products. Regul Toxicol Pharmacol.

[15] Lim CC, Peng IC, Huang YH (2020). Safety of intrastromal injection of polyhexamethylene biguanide and propamidine isethionate in a rabbit model. J Adv Res.

[16] Clarke DW, Alizadeh H, Niederkorn JY (2005). Failure of Acanthamoeba castellanii to produce intraocular infections. Invest Ophthalmol Vis Sci.

[17] Moshari A, McLean IW, Dodds MT, Damiano RE, McEvoy PL (2001). Chorioretinitis after keratitis caused by Acanthamoeba: case report and review of the literature. Ophthalmology.

[18] Arnalich-Montiel F, Martín-Navarro CM, Alió JL (2012). Successful monitoring and treatment of intraocular dissemination of acanthamoeba. Arch Ophthalmol.

[19] Mammo Z, Almeida DR, Cunningham MA, Chin EK, Mahajan VB (2017). Acanthamoeba endophthalmitis after recurrent keratitis and nodular scleritis. Retin Cases Brief Rep.

[20] Raghavan A, Veerappan S, Rangarajan V (2020). Fulminant Acanthamoeba endophthalmitis after cataract surgery - a case report. Cornea.

